# Phenological responses to climate change based on a hundred years of herbarium collections of tropical Melastomataceae

**DOI:** 10.1371/journal.pone.0251360

**Published:** 2021-05-07

**Authors:** Duane F. Lima, José H. F. Mello, Isadora T. Lopes, Rafaela C. Forzza, Renato Goldenberg, Leandro Freitas

**Affiliations:** 1 Jardim Botânico do Rio de Janeiro, Rio de Janeiro, Brazil; 2 Departamento de Botânica, Universidade Federal do Paraná, Curitiba, Paraná, Brazil; Universite du Quebec a Chicoutimi, CANADA

## Abstract

Changes in phenological events have been vastly documented in face of recent global climate change. These studies are concentrated on temperate plants, and the responses of tropical species are still little understood, likely due to the lack of long-term phenological records in the tropics. In this case, the use of herbarium specimens to gather phenological data over long periods and wide geographic areas has emerged as a powerful tool. Here, we used four Melastomataceae species endemic to the Brazilian Atlantic Forest to evaluate phenological patterns and alterations as responses to recent climate changes. Phenological data were gathered from Reflora Virtual Herbarium specimens collected between 1920 and 2018, and analyzed with circular statistics applied to the intervals 1920–1979, 1980–1999, and 2000–2018. The effects of temperature range, average temperature, precipitation, and photoperiod on flowering and fruiting of each species were tested using multiple linear regressions. Through circular statistics, we detected changes, mostly delays, in the flowering of *Miconia quinquedentata*, *Pleroma clavatum* and *P*. *trichopodum*, and in the fruiting of *M*. *acutiflora*, *P*. *clavatum* and *P*. *trichopodum*. We also found that flowering and fruiting occurrence were related to local climatic conditions from months prior to the collections. We found marked phenological variations over the decades and also that these variations are associated to global climate change, adding up to the large body of evidence from higher latitudes. Our results also support herbarium collections as an important source for long-term tropical phenological studies. The lack of consistent patterns of responses among the four species (e.g. fruiting delayed two months in *P*. *clavatum* and advanced one month in *M*. *acutiflora*) suggests that climate change has unequal effects across tropical forests. This highlights the urgent need for further research to understand and forecast the ecological implications of these changes in global ecosystems processes.

## Introduction

Phenology can be summarized as the study of the timing of life cycle events, such as production of flowers and fruits [1]. These events are commonly triggered by environmental cues, and therefore well-suited to understand how temporal and spatial variation may affect plant life cycle [2]. In recent years, the use of phenological data to predict species responses to global climate change is steadily becoming an effective tool in conservation science [3], particularly in the temperate northern hemisphere. The seasonal weather in this region leads to a well-marked reproductive period (generally following warmer temperatures and longer day lengths) followed by a resting period (generally along colder temperatures and shorter day lengths), suggesting that in these areas, temperature and photoperiod play major roles in phenology [4]. This evident pattern facilitates the long-term monitoring of phenology, a crucial point to analyze how climatic variations are affecting plant life cycle [5]. For instance, several studies have shown that temperate plants are flowering earlier with global warming [e.g. 6,7], but delayed flowering has also been reported [e.g. 8].

On the other hand, the weather in the tropical region is more stable throughout the year. This, allied to the immense tropical plant species diversity, makes the understanding of phenological patterns more complex and challenging [9]. In the tropical forests, precipitation and photoperiod have been indicated as important factors regulating reproductive phenology of plants, while temperature seems to be less crucial [10–12]. Nevertheless, understanding how tropical species respond to global climatic change is still a large gap in ecological and botanical knowledge. Surveys on this topic are scarce in the tropics, likely due to the lack of long-term phenological records, and to deficient historical climatic data [2,13]. Studies that rely on direct evidence and statistical tests are even rarer. Although not always clearly, some of these studies have found changes in timing or intensity of flowering and fruiting, mainly in response to alterations in temperature and rainfall, in tropical regions of China [14], Africa [15,16], and Central America [17]. Nevertheless, lack of specific correlation between fruiting and rainfall was also found in an African tropical rainforest [18]. More specifically in Brazil, data from meteorological stations are widely fragmented [see 19], making it difficult to be consistently used in large scales. To our knowledge, a single study explicitly tested phenological responses related to global climate change in that country and found no significant variations in flowering and fruiting of two species of *Leptolobium* (Fabaceae) in a time frame of 52 years [20].

Natural history collections have emerged as a powerful tool when long phenological records *in loco* are not available [21]. Herbaria shelter data not only on taxonomic and phylogenetic diversity of plants, but also on ecological aspects such as species distribution and phenophases along both space and time. Although in some cases relatively incomplete, herbarium data generally spam long temporal and spatial scales, which may allow inference of species responses to environmental changes [2]. Fortunately, recent advances in high-resolution imagery technology, coupled with Geographic Information Systems (GIS), database integration, and improvements in analytical frameworks of herbarium collections have renewed interest in the field [21].

The Reflora Virtual Herbarium (RVH, http://reflora.jbrj.gov.br), is an herbarium digitalization initiative that had conservation as a primary objective since its early conception [22]. Established by the Brazilian Federal government in 2010, the RVH main goal is to make publicly available collection data and high-resolution images of Brazilian exsiccates deposited both in Brazil and abroad. The RVH currently has >3.7 million images of plant specimens deposited in 86 herbaria in Brazil, United States and Europe [23], representing 93% of the flowering plant species and 100% of genera and families found in all Brazilian territory [22]. Melastomataceae was chosen for the phenological studies proposed here because it is quite well represented in RVH with approximately 131,000 digitalized images readily available, many of them confidently identified by taxonomists working on the group. This is one of the most diverse families in Brazil [24], comprising 69 genera and 1436 species occurring in all Brazilian biomes, of which 929 are considered endemic [25]. Hitherto, a few studies on phenology of Melastomataceae species have been done [but see 26–29], none using exclusively herbarium data, nor linking phenology and historical environmental changes.

Within this context, our study aims to evaluate tropical plant responses to climate changes during the last one hundred years. For that, we adopted four Melastomataceae species from the Brazilian Atlantic Forest as model. The main objectives were: i) to assess phenological patterns of these species based on data from the RVH, and their relation to climate conditions, and ii) to examine whether phenological patterns have changed through the years.

## Materials and methods

### Species selection

Four basic criteria were considered during species selection. First, species must have clearly identifiable reproductive structures (flowers and fruits), in order to facilitate structure visualization via images; second, species must have at least 200 registers in RVH (including their synonyms according to [25]); third, species should be restricted to a single biome, in order to facilitate analyses and comparisons; fourth, species should be from different Melastomataceae tribes, in order to evaluate a potentially broader scope of responses to climate changes given the differences in flower and fruit morphologies (see [26]). As a result, four species with distribution restricted to the Atlantic Forest biome were chosen: *Miconia acutiflora* (Naudin) R.Goldenb., *M*. *quinquedentata* (DC.) R.Goldenb., *Pleroma clavatum* (Pers.) P.J.F.Guim. & Michelang. and *P*. *trichopodum* DC. (species names follow [30,31]). The former two species belong to tribe Miconieae, presenting small white or light-yellow flowers and fleshy fruits, while the others belong to tribe Melastomateae and present large flashy-colored flowers and dry fruits.

### Specimens and phenological data gathering

For all selected species (including their synonyms), we obtained collection records directly from RVH in August 2018, from specimens that had been collected between 1920 and 2018. Each dataset was then thoroughly verified in order to remove duplicates (i.e. specimens with exact same collector, date and locality), sterile specimens, and specimens without collection date or locality (whenever this information was missing in the database, we sought the image of the material to verify whether they were available in the exsiccate label or not). We also removed all specimens that had dubious identification at species level by visualizing the images of the specimens and excluding those ones with discrepant morphologies.

For each species, the data cleaning process resulted in datasets comprised by collectors’ name and number, collection date, collection locality and geographic coordinates. Roughly, 50% of specimens were removed from each species dataset during data cleaning (as described above), as follows: *Miconia acutiflora*, 374 initial entries (IE) and 196 in the final dataset (FD); *M*. *quinquedentata*, 369 IE and 183 FD; *Pleroma clavatum*, 336 IE and 102 FD; and *P*. *trichopodum*, 359 IE and 186 FD. Each specimen was scored according to its reproductive phenophase: i) flowering, i.e., presence of open flowers; and/or ii) fruiting, i.e., presence of fruits. This implies that the presence of flowers/fruits in each specimen in a given date (date of collection) actually represents any time of the flowering/fruiting period of such species, between the beginning and the end of the phenophase. The bulk of specimens provides a good picture of the time, duration, and peak of flowering and fruiting of each species [e.g. 7]. To achieve consistency, a sufficient sample size is necessary; because of that, we selected only species with large numbers of specimens available in RVH.

### Seasonality analyses

Phenological studies deal with cyclic biological events, and therefore are not easily analyzed in linear scales. The rationale is that, because this kind of data presents periodicity, it should either be analyzed with time-series analyses, which add several levels of complexity to it, or in circular scales, which are comprised in an interval between 0° and 360° (0~2π, in radians) [32]. In this case, each month corresponds to a 30° arc in a year that represents a 360° circle (January = 0°, February = 30°, …, December = 330°). Circular statistics allow a much more treatable evaluation of phenological data and have been recommended for analyses of phenology in the tropics [5,33]. In order to explore possible variations in seasonality between different time intervals, circular statistics were applied to three datasets for each species: i) interval I—specimens collected between 1920–1979; ii) interval II—specimens collected between 1980–1999; and iii) interval III—specimens collected between 2000–2018. The long span (60 years) in interval I was due to the lower number of collections from that period. The number of specimens in each dataset is presented in S1 Table.

One of the main issues while dealing with circular data is deciding which test of uniformity is the best to apply. Most published works evaluate directionality by applying Rayleigh’s test [e.g. 29,34], which assumes that data is unimodal with a Von Mises underlying distribution. However, as showed by Landler et al. [33], the statistical power of this particular test drops steeply when data is not unimodal. These authors’ results show that, when dealing with non-axial (i.e., asymmetrical) multimodal distributions, the Hermans-Rassons (HR) test outperforms Rayleigh’s test for assessing deviations from circular homogeneity. Therefore, we followed a maximum likelihood-based (MLE) approach to determine which model presented the best fit for each dataset. This MLE approach was first proposed by Schnute and Groot [35], having been recently implemented by Fitak and Johnsen [36] in the package *CircMLE* for the R environment [37]. For each species, all 10 models described by Schnute and Groot [35] were tested for each time interval. Pseudo-replication was not an issue since each specimen was independently collected. Based on the MLE results, we then proceeded to assess which, if any, datasets presented a preferred direction, by applying the adequate tests: Rayleigh’s test for unimodal distributions and Hermans-Rasson’s test for multimodal distributions [33].

Following the circular analyses, we evaluated if there were any significative variations between time intervals via Watson’s U^2^ test for grouped data [32]. The choice for Watson’s U^2^ test was based on the MLE analyses, which showed that some of the datasets fitted best the multimodal distributions. Watson’s U^2^ is a non-parametric test that presents a relative flexibility regarding circular data with different distributions. Only the basic form of this test is currently implemented in R in the package *circular* [38], and therefore we developed the code used for this modification (S1 File).

### Climatic data and analyses

Climatic data were obtained from CHELSAcruts data series at 30 arc-seconds resolution [39]. CHELSA is a climate dataset with estimates of mean monthly maximum and minimum temperatures, and monthly precipitation sums. Because CHELSA comprises data from 1901 to 2016, we exceptionally excluded phenological records from 2017 and 2018 in this analysis. For each specimen, raster values for these variables, matching year, month, and locality were obtained in R environment with packages *raster* [40] and *rgdal* [41]. Coordinates of municipality centroids where each specimen was collected were assessed in QGIS 3.8.2 [42] with *realcentroid* plugin. Mean monthly temperatures and temperature range were assessed based on maximum and minimum temperatures for each specimen. Additionally, photoperiod data were obtained with package *suncalc* [43] in R. To illustrate climatic changes over the study period, mean annual maximum and minimum temperatures, and mean annual precipitation from both the northern- and southern-most geographic coordinates of our sampling (18°18’3’’S–39°57’25’’W and 30°21’19’’S–51°18’52’’W, respectively) were plotted into graphs. Additionally, we used One-way ANOVA to determine if those climatic variables varied among the time intervals I (1920–1979), II (1980–1999) and III (2000–2016).

In order to explore how phenological data were related to variation in climatic elements, we built, for each species in each phenophase (flowering and fruiting), multiple regression models using the angle of the month of collection (in radians) as the dependent variable and climatic data extracted from each geographic coordinate as the independent variable. The climatic variables analyzed were mean monthly precipitation, mean monthly temperature range, mean monthly temperature and photoperiod. We included climatic data from the month of the collection and also from one, two and three months prior to the collection. Before building the regression models, we evaluated potential collinearity of our environmental variables both via Pearson’s correlation coefficient and by variance-inflation factors using package *car* [44] in R. The full models included all climatic variables and time lags. To build the final regression models, we performed a stepwise backwards model selection, removing all non-informative variables from the full models using package MASS [45] in R.

Finally, *Pleroma clavatum* and *P*. *trichopodum* were used to compare how sensible climatic analyses are regarding the accuracy of available geographic information, since the climatic data gathered using municipality centroids may not reflect the precise meteorological conditions where the plants occur. In this case, the same analyses as described above were done, but otherwise using the exact coordinates where collections took place. For the vast majority of specimens, the exact coordinate was not available on the specimen label and was manually searched on Google Earth.

## Results

**Fig 1:**
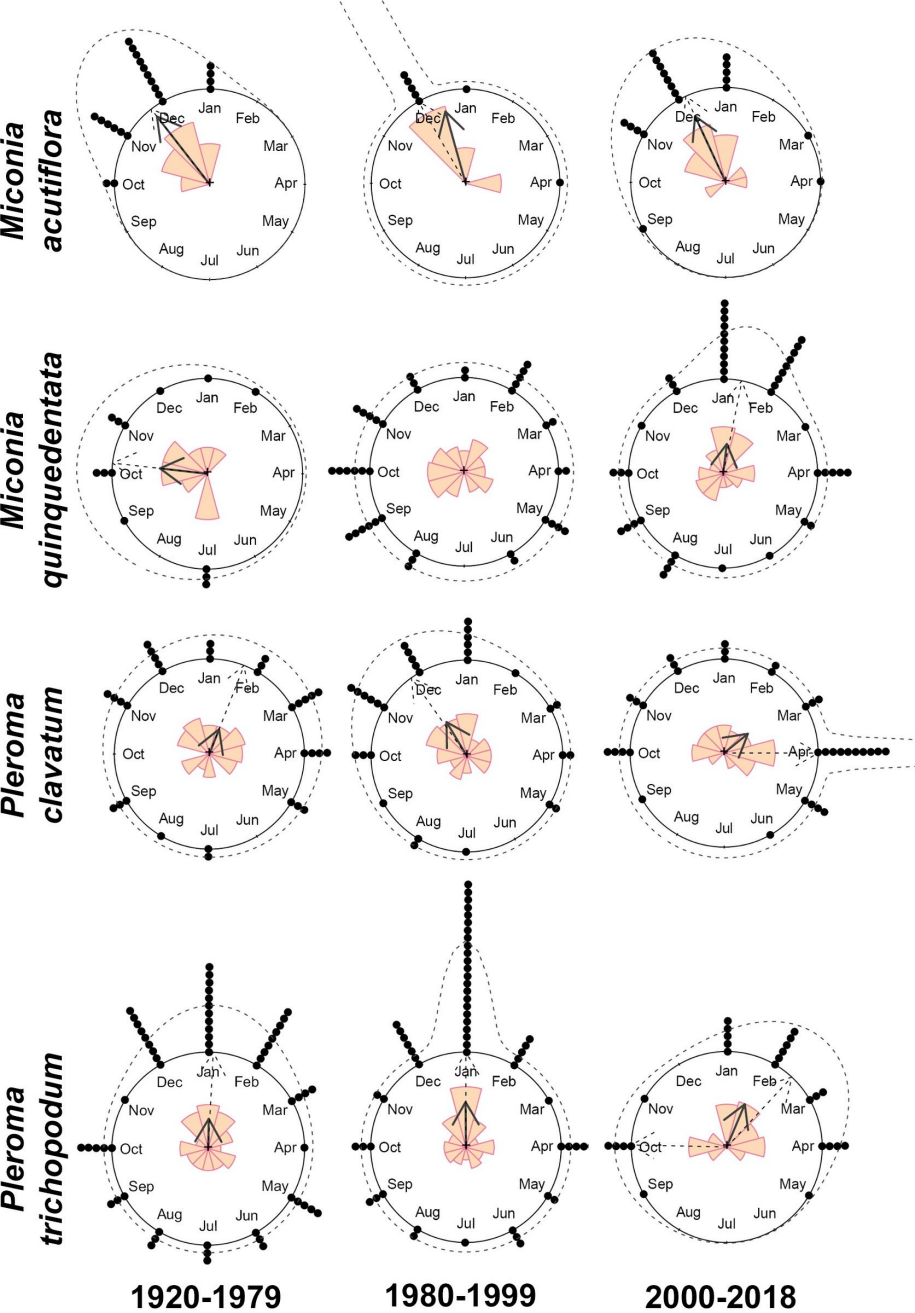
Circular distribution of flowering specimens of *Miconia acutiflora*, *M*. *quinquedentata*, *Pleroma clavatum* and *P*. *trichopodum* over the year. Observed and modelled circular distributions of flowering events for the three time intervals (1920–79, 1980–99, and 2000–18). The observed mean direction is shown as solid grey arrows, circular histograms (radii equals square root of relative frequencies) as colored bars, samples as black dots, modelled directions as dashed arrows, and modelled density distribution as dashed lines.

**Fig 2:**
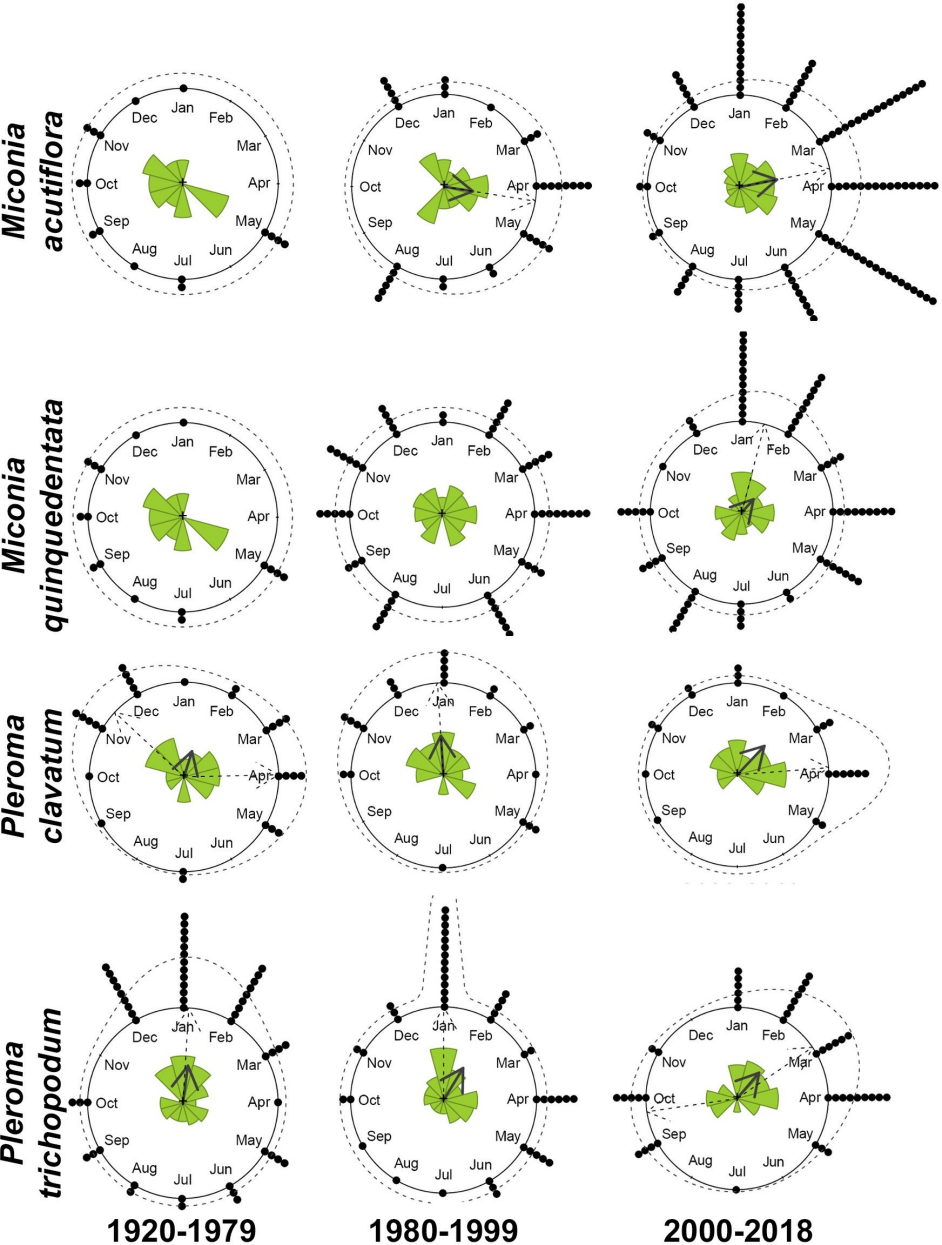
Circular distribution of fruiting specimens of *Miconia acutiflora*, *M*. *quinquedentata*, *Pleroma clavatum* and *P*. *trichopodum* over the year. Observed and modelled circular distributions of fruiting events for the three time intervals (1920–79, 1980–99, and 2000–18). The observed mean direction is shown as solid grey arrows, circular histograms (radii equals square root of relative frequencies) as colored bars, samples as black dots, modelled directions as dashed arrows, and modelled density distribution as dashed lines.

Considering all specimens together, the four studied species were registered with flowers and fruits throughout the year, except *Miconia acutiflora* that had no flowering records between May and August. *Pleroma trichopodum* had flowered specimens every month but concentrated between December and February. In turn, fruiting specimens were recorded more evenly along the year in all species (S1 Fig). Circular statistics of *M*. *acutiflora*, *M*. *quinquedentata*, *P*. *clavatum* and *P*. *trichopodum* through the time intervals, as well as mean dates of flowering and fruiting, are summarized in Table 1 and Figs 1 and 2. Detailed results from the MLE analyses are presented in S2 Table. Lastly, results from the Watson’s U^2^ tests, comparing the time intervals are shown in Table 2.

**Table 1:** Results of circular analyses for the occurrence of seasonality in flowering and fruiting of *Miconia acutiflora*, *M*. *quinquedentata*, *Pleroma clavatum* and *P*. *trichopodum* during the three time intervals (1920–79, 1980–99, 2000–18).

Species	Time interval	Best-fit model	Distribution	Test	Test statistics	p	Mean vector length (*r*)	Mean angle (radians)	Kappa	Lambda	Mean date (day/month)
***Flowering***											
*Miconia acutiflora*	1920–1979	M2A	unimodal	Rayleigh’s	0.9022	0	0.90	5.622	5.417	1.000	26/Nov
	1980–1999	M2B	unimodal	Rayleigh’s	0.791	0.007	0.79	6.009	227.000	0.500	16/Dec
	2000–2018	M2A	unimodal	Rayleigh’s	0.7678	0	0.77	5.849	2.526	1.000	07/Dec
*Miconia quinquedentata*	1920–1979	M2A	unimodal	Rayleigh’s	0.5004	0.035	0.50	4.825	1.161	1.000	08/Oct
	1980–1999	M1	uniform	Rayleigh’s	0.2105	0.17	NA	NA	NA	NA	NA
	2000–2018	M2C	unimodal	Rayleigh’s	0.315	0.011	0.32	0.125	11.916	0.348	09/Jan
*Pleroma clavatum*	1920–1979	M2A	unimodal	Rayleigh’s	0.2788	0.076	0.28	0.375	0.581	1.000	-
	1980–1999	M2B	unimodal	Rayleigh’s	0.4128	0.004	0.41	5.741	2.943	0.500	01/Dec
	2000–2018	M2C	unimodal	Rayleigh’s	0.3254	0.026	0.33	0.890	195.234	0.250	22/Feb
*Pleroma trichopodum*	1920–1979	M2B	unimodal	Rayleigh’s	0.3085	0.004	0.31	0.018	4.428	0.500	02/Jan
	1980–1999	M2C	unimodal	Rayleigh’s	0.4748	0	0.47	6.275	130.182	0.320	31/Dec
	2000–2018	M5A	bimodal non-axial	Hermans-Rasson	72.3523	0.001	NA	0.758	3.237	0.749	14/Feb 03/Oct
								4.731	3.237	0.251	
***Fruiting***											
*Miconia acutiflora*	1920–1979	M4A	bimodal axial	Hermans-Rasson	69.097	0	NA	5.720	3.448	0.691	29/Nov 31/May
								8.862	3.448	0.309	
	1980–1999	M2A	unimodal	Rayleigh’s	0.316	0.029	0.32	1.728	0.667	1.000	11/Apr
	2000–2018	M2A	unimodal	Rayleigh’s	0.416	0	0.42	1.375	0.916	1.000	22/Mar
*Miconia quinquedentata*	1920–1979	M1	uniform	Rayleigh’s	0.288	0.27	NA	NA	NA	NA	NA
	1980–1999	M1	uniform	Rayleigh’s	0.081	0.69	NA	NA	NA	NA	NA
	2000–2018	M2C	unimodal	Rayleigh’s	0.196	0.065	0.20	0.721	6.796	0.250	-
*Pleroma clavatum*	1920–1979	M5A	bimodal non-axial	Hermans-Rasson	88.389	0.043	NA	1.548	2.361	0.506	01/Apr 15/Nov
								5.463	2.361	0.494	
	1980–1999	M2A	unimodal	Rayleigh’s	0.428	0.013	0.43	6.219	0.950	1.000	28/Dec
	2000–2018	M2B	unimodal	Rayleigh’s	0.432	0.018	0.43	0.757	8.939	0.500	14/Feb
*Pleroma trichopodum*	1920–1979	M2B	unimodal	Rayleigh’s	0.395	0	0.40	0.136	5.453	0.500	09/Jan
	1980–1999	M2C	unimodal	Rayleigh’s	0.403	0.001	0.40	0.549	104.700	0.275	02/Feb
	2000–2018	M5A	bimodal non-axial	Hermans-Rasson	132.375	0	NA	0.974	2.704	0.732	26/Feb 23/Sep
								4.566	2.704	0.268	

The application of Rayleigh’s or Hermans-Rasson test is according to the best-fit model of data distribution for each dataset (see Materials and methods). Mean angle and mean date are omitted when not significant.

**Table 2:** Watson’s U^2^ values between time intervals for each species.

*Miconia acutiflora*			
	1920–1979	1980–1999	2000–2018
1920–1979	-	0.1056	0.0341
1980–1999	0.1841	-	0.0893
2000–2018	**0.3715**	0.0732	-
***Miconia quinquedentata***			
	1920–1979	1980–1999	2000–2018
1920–1979	-	0.0706	**0.2709**
1980–1999	0.1165	-	**0.216**
2000–2018	**0.2796**	0.1107	-
***Pleroma clavatum***		
	1920–1979	1980–1999	2000–2018
1920–1979	-	0.0834	0.0834
1980–1999	0.0557	-	**0.1951**
2000–2018	**0.0446**	0.1059	-
***Pleroma quinquedentata***			
	1920–1979	1980–1999	2000–2018
1920–1979	-	0.1249	0.1285
1980–1999	**0.0631**	-	0.1581
2000–2018	0.2173	**0.1221**	-

Flowering above and fruiting below diagonal. Bold values = p < 0.05.

Considering flowering phenology only, *M*. *acutiflora* was better fitted under unimodal distribution in all intervals, with mean flowering date remaining relatively the same between late November and middle December (Tables 1 and 2; Fig 1). *Miconia quinquedentata* changed from unimodal distribution in interval I with mean date in early October to uniform distribution in interval II (no mean date), and reversed to unimodal in interval III, but with a delay to early January; changes between intervals I and III, and II and III were significant (Tables 1 and 2, Fig 1). *Pleroma clavatum* flowering presented no significant directionality in interval I but did in interval II with mean date in early December, and interval III with delayed mean date to late February (Tables 1 and 2, Fig 1). *Pleroma trichopodum* presented unimodal distributions in both intervals I and II with mean dates remaining roughly the same, in early January and late December, respectively. A transition towards bimodal pattern in interval III was observed with a delayed first peak in early February and a second peak in early October, but this last change was not significant (Tables 1 and 2, Fig 1).

Regarding fruiting distribution, all species presented variations among time intervals. *Miconia acutiflora* had a bimodal distribution in interval I with mean dates in late November and late May. The first peak was apparently lost in the following intervals, when there was a change to unimodal distribution with similar mean dates (around late March and middle April); the change between interval I and III was significant (Tables 1 and 2, Fig 2). *Miconia quinquedentata* was better fitted under uniform distribution in intervals I and II. In the subsequent interval, the species changed to a unimodal pattern, but Rayleigh’s test did not show a significant direction (Table 1, Fig 2), showing that fruiting remained mostly dispersed year-round. *Pleroma clavatum* switched from bimodal in interval I with mean dates in early April and middle November to a unimodal distribution in interval II that continued in the following interval. A delay in fruiting from late December to early February was also observed. Only the change between intervals I and III was significant (Tables 1 and 2, Fig 2). Finally, *P*. *trichopodum* had unimodal distributions in the first two intervals with mean dates in early January in interval I and a delay to early February in interval II. The interval III had a change to bimodal distribution. The first peak was further delayed to late February, and an additional peak appeared in late September. Alterations were significant between intervals I and II, and I and III (Tables 1 and 2, Fig 2).

**Fig 3:**
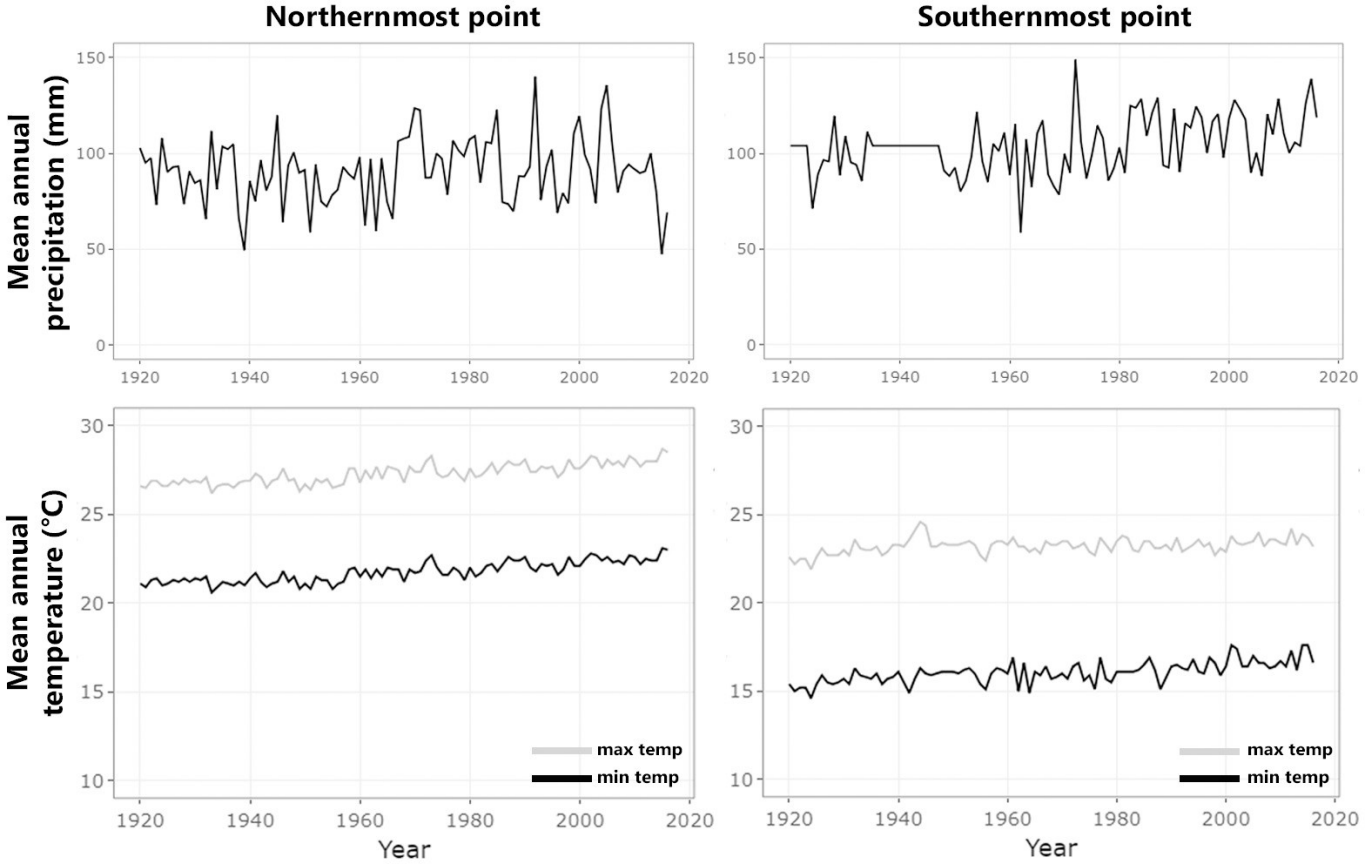
Mean annual precipitation and temperature (minimum and maximum) between 1920 and 2016 in the northern- and southernmost points (geographic coordinates) sampled in this study. Climatic data were obtained from CHELSAcruts data series. The northern- and southern-most points are 18°18’3’’S–39°57’25’’W and 30°21’19’’S–51°18’52’’W, respectively. Mean annual precipitation presented great variation between years, while both minimum and maximum temperatures constantly increased along the time interval analyzed.

While the mean annual temperatures seem to increase constantly, the mean annual precipitation looks more erratic between years during the study period (1920–2016; Fig 3, see details in S2 Fig). Overall, variations in the minimum and maximum temperatures and in the precipitation among time intervals were significant, except the precipitation in the northernmost point (S3 Table).

Flowering and fruiting of all species were associated to local climatic conditions (Table 3), which explained phenological variation from 72% to 86% (R^2^; p < 0.001). The best models included different combinations of the climatic variables (photoperiod, precipitation, average temperature, and temperature range) from the month of sampling and from the three previous months. For instance, the best model for fruiting of *M*. *acutiflora* included all climatic variables and different time lags, while flowering of *P*. *clavatum* was best explained by photoperiod and average temperature variation, also combining different time lags (Table 3). Fruiting of *P*. *clavatum* was the only event that was best explained by a single independent variable (photoperiod), but also combining different time lags (Table 3). Overall, photoperiod variation explained reproductive phenology more than any other climatic element. Results of climate analyses using the exact coordinates of specimens of *P*. *clavatum* and *P*. *trichopodum* were highly similar to those using municipalities centroids (S4 Table).

**Table 3:** Summary of the multiple linear regression analysis for climatic variables predicting flowering and fruiting phenology of *Miconia acutiflora*, *M*. *quinquedentata*, *Pleroma clavatum* and *P*. *trichopodum*.

Species	Summary	Climatic variable	Slope	SE	t-value	p-value
***Miconia acutiflora***	**Flowering**					
	R^2^ = 0.812 F = 26.39 N = 48 p < 0.001	*Intercept*	77.587	15.284	-5.076	<0.001
		Photoperiod (0)	0.296	0.047	6.251	<0.001
		Precipitation (-1)	-0.009	0.003	-2.846	0.007
		Temperature range (-1)	-0.034	0.01	-3.31	0.002
		Photoperiod (-1)	-0.324	0.056	-5.809	<0.001
		Temperature range (-3)	0.03	0.011	2.683	0.011
		Photoperiod (-3)	0.14	0.029	4.828	<0.001
	**Fruiting**					
	R^2^ = 0.834 F = 73.62 N = 159 p < 0.001	*Intercept*	31.626	6.36	-9.69	<0.001
		Temperature range (0)	-0.018	0.005	-3.625	<0.001
		Photoperiod (0)	0.226	0.024	9.578	<0.001
		Precipitation (-1)	-0.004	0.001	-4.606	<0.001
		Average temperature (-1)	-0.027	0.008	-3.537	<0.001
		Photoperiod (-1)	-0.194	0.048	-4.067	<0.001
		Precipitation (-2)	0.003	0.001	3.3	0.001
		Average temperature (-2)	0.024	0.008	3.074	0.002
		Temperature range (-2)	0.011	0.005	2.111	0.036
		Photoperiod (-2)	-0.103	0.048	-2.139	0.034
		Photoperiod (-3)	0.161	0.024	6.804	<0.001
***Miconia quinquedentata***	**Flowering**					
	R^2^ = 0.854 F = 63.64 N = 96 p < 0.001	*Intercept*	63.967	7.277	-8.79	<0.001
		Photoperiod (0)	0.306	0.029	10.354	<0.001
		Precipitation (-1)	-0.002	0.001	-2.373	0.02
		Photoperiod (-1)	-0.402	0.065	-6.23	<0.001
		Average temperature (-2)	0.02	0.01	2.124	0.036
		Average temperature (-3)	-0.035	0.01	-3.625	<0.001
		Photoperiod (-3)	0.083	0.03	2.728	0.008
	**Fruiting**					
	R^2^ = 0.862 F = 99.91 N = 142 p < 0.001	*Intercept*	70.534	6.245	-11.295	<0.001
		Temperature range (0)	-0.02	0.005	-3.673	<0.001
		Photoperiod (0)	0.339	0.023	14.442	<0.001
		Precipitation (-1)	-0.004	0.001	-4.065	<0.001
		Photoperiod (-1)	-0.451	0.048	-9.418	<0.001
		Photoperiod (-2)	0.019	0.005	3.71	<0.001
		Average temperature (-3)	0.119	0.048	2.469	0.015
		Photoperiod (-3)	0.094	0.024	3.981	<0.001
***Pleroma clavatum***	**Flowering**					
	R^2^ = 0.723 F = 43.88 N = 95 p < 0.001	*Intercept*	64.185	10.659	-6.021	<0.001
		Photoperiod (0)	0.282	0.035	8.044	<0.001
		Average temperature (-1)	-0.029	0.014	-2.066	0.042
		Photoperiod (-1)	-0.324	0.041	-7.909	<0.001
		Average temperature (-2)	0.028	0.013	2.142	0.035
		Photoperiod (-3)	0.131	0.02	6.47	<0.001
	**Fruiting**					
	R^2^ = 0.778 F = 59.6 N = 68 p < 0.001	*Intercept*	-67.02	11.672	-5.742	<0.001
		Photoperiod (0)	0.293	0.038	7.765	<0.001
		Photoperiod (-1)	-0.341	0.044	-7.765	<0.001
		Photoperiod (-3)	0.144	0.022	6.606	<0.001
***Pleroma trichopodum***	**Flowering**					
	R^2^ = 0.808 F = 71.6 N = 135 p < 0.001	*Intercept*	-74.39	8.156	-9.121	<0.001
		Temperature range (0)	-0.012	0.004	-2.768	0.006
		Photoperiod (0)	0.372	0.032	11.546	<0.001
		Photoperiod (-1)	-0.52	0.063	-8.273	<0.001
		Photoperiod (-2)	0.164	0.06	2.778	0.006
		Precipitation (-3)	-0.003	0.001	-2.026	0.045
		Photoperiod (-3)	0.095	0.028	3.425	<0.001
	**Fruiting**					
	R^2^ = 0.797 F = 59.5 N = 135 p < 0.001	*Intercept*	70.264	7.84	-8.962	<0.001
		Temperature range (0)	-0.012	0.004	-2.955	0.004
		Photoperiod (0)	0.339	0.031	10.99	<0.001
		Photoperiod (-1)	-0.458	0.058	-7.912	<0.001
		Precipitation (-2)	0.002	0.001	2.445	0.016
		Photoperiod (-2)	0.119	0.053	2.264	0.025
		Precipitation (-3)	-0.003	0.001	-2.242	0.027
		Photoperiod (-3)	0.104	0.025	4.149	<0.001

Slopes, standard errors (SE), *t* and *p* values of each climatic variable in the final regression models, i.e. after removal of non-significant variables, for data on flowering and fruiting phenology. Time lags are in parenthesis after each climatic variable.

## Discussion

In this study, we aimed to evaluate reproductive phenological changes of four Melastomataceae species endemic to the Brazilian Atlantic Forest along nearly 100 years, and how these changes can possibly be related to the global climate change. Using circular statistics applied to three time intervals (1920–1979, 1980–1999 and 2000–2018) for each species, we did detect significant alterations for flowering and fruiting phenology of *Miconia acutiflora*, *M*. *quinquedentata*, *Pleroma clavatum* and *P*. *trichopodum*. Concomitantly, temperature continuously increased and precipitation varied greatly between 1920 and 2016 in the Atlantic Forest, considering the data of the latitudinal limits sampled in this study (Fig 3; see details in S2 Fig). Although circular statistics have been widely used in phenological field studies in the Neotropics [e.g. 26,34], to our knowledge this approach was not employed before to examine variations in phenology through long time series.

When comparing the results of circular statistics, phenological changes were evident: the timing of flowering of *M*. *quinquedentata*, flowering and fruiting of *P*. *clavatum*, and fruiting of *P*. *trichopodum* were clearly delayed; fruiting of the last two species had also changed their seasonality patterns (from bimodal to unimodal or vice-versa); and lastly, fruiting of *M*. *acutiflora* advanced. It has been reported that phenological responses to climate change in the tropics vary largely among species, localities, and phenophases [46]. For instance, species from tropical China were found to flower earlier over the years [14], while species from tropical South America tend to have delayed flowering [20]. In contrast, several studies have shown that temperate species present in general a similar trend of earlier production of reproductive structures following global warming [e.g. 6,7,47].

All these historical alterations may have huge biological impact at both populational and community levels [48], for instance affecting the reproduction of self-incompatible species, which require an overlap in flowering time among individuals or even populations, or decoupling plant-pollinator and plant-disperser interactions [49,50]. Limited pollination directly impacts reproductive success, consequently decreasing genetic diversity and population viability, and may even compromise species survival [51, see also 52]. In the Brazilian Atlantic Forest, niche models have shown that both geographical distribution and flowering phenology of plant species are prone to be altered under climate change, consequently impacting their occurrence, reproductive success, and ecological networks [53]. Besides compromising plant reproduction, flowering changes can also directly impact the pollinator life cycle. For instance, bumblebees’ interannual abundance is driven by temporal distribution of floral resources, which in turn has been affected by climate change [54]. This seems of key importance in cases where interannual flowering seasonality changes because it affects the populational dynamics of the pollinator along the year. Moreover, entire communities could be reshaped following shifts in plant reproductive phenology, for example where coflowering patterns within the community are altered [55]. Particularly in the case of *Miconia* species, their fruits and seeds are usually dispersed by a bunch of frugivorous [56], and temporal alterations in the production of fruits and seeds may also impact animals that depend on it for food [50], likely leading to trophic mismatches and cascading effects on communities [46], in spite of possible network resilience by rewiring.

Our regression results corroborate the evidence that reproductive phenology of tropical plants is related by abiotic factors, such as photoperiod (day length) and temperature [e.g. 10]. The photoperiod particularly seems to play an important role in the plant reproductive phenology in the Atlantic Forest [10,57,58], which comprises a mosaic of seasonal and non-seasonal environments with different climate regimes [59]. Our regression models have benefit from the inclusion of climatic data from the previous months of collections, which is consistent with expectations for moderate-acting ecophysiological responses. Specifically, photoperiod is a reliable signal for temporal cycles even in regions with low seasonality [10,57,58,60]. In tropical forests from higher latitudes as the Atlantic Forest, annual variation in photoperiod anticipates movements of the intertropical convergence zone and consequent seasonal changes in precipitation, irradiance, and biotic activity [61]. However, the combination of different variables and time lags in our best regression models highlights that the physiological regulation of flowering and fruiting by climate in the Atlantic Forest is complex, with each species responding singularly. Because of that, there are always different species producing flowers or fruits at any time of a year, making interpretation of tropical plant responses harder when compared to temperate species [9,46]. This implies, at least partially, that projections of phenological responses to global warming are more complicated for tropical vegetation. In fact, some models have predicted that, in face of climate impacts, temperate species tend to shift their phenology timing, while tropical species tend to change their geographical distribution, or to evolve [62].

Overall, when considering all specimens together (from 1920 to 2018), flowering was continuous throughout the year in *M*. *quinquedentata* and *P*. *clavatum*. In contrast, *M*. *acutiflora* had a clear annual pattern, with a peak from November to January, while *P*. *trichopodum* flowered along the whole year but concentrated between December and February (S1 Fig). Studies based on field observations of a set of Melastomataceae species from specific areas of Atlantic Forest [26] found differences in flowering strategies depending on the pollination system: it was aseasonal in pollinator-independent species, while pollinator-dependent species presented some degree of seasonality. Although our sampling is not adequate to test the validity of these associations, some similarities can be observed, as *P*. *clavatum* and *P*. *trichopodum* are respectively independent [26] and dependent on pollinators [63]. Regarding fruits, the four species sampled here fruited continuously along the year, again corroborating field observations that reported the same pattern, even for species with seasonal flowering [26]. Despite sampling constraints, our results validate the use of herbarium specimens for phenological studies in Melastomataceae.

This was the first study based exclusively on biological and geographic data publicly available on the RVH for phenological purposes, reinforcing the utility of these rich Brazilian database as a substitute when field observations are inexistent. Natural history collections have been proved to be suitable for phenological research in animals [64] and plants [21]. However, though easily available, dealing with large data from collections is not an easy task. The RVH centralizes data from various herbaria and, as a result, it also includes errors (mainly date and geographic incomplete information). In our dataset, for example, several specimens had no dates transcribed from the exsiccate label to the RVH database, highlighting the importance of a careful data curation in order to get the most of these online databases. Finally, the use of herbarium collections on tropical phenological research is still rare [65]. Within this scenario, the approach employed here points to new possibilities to explore tropical terrestrial ecosystem feedback to the ongoing climate change.

## Conclusions

In this work, the large number of digitalized herbarium specimens from the RVH allowed us to create a solid phenological dataset of four Melastomataceae species, spanning a wide geographic area (the Brazilian Atlantic Forest) and a long time interval (nearly 100 years). By analyzing >650 herbarium specimens with multiple statistical tests, we detected changes in both timing and seasonality of flowering and fruiting over the decades, which are likely to cause direct biological impacts in populations (gene flow) and communities (trophic interactions). Also, as reported in previous studies, we found that reproductive phenology is strongly tied to local climatic conditions. Given the recent climatic anomalies such as warming temperatures, we hypothesize that flowering and fruiting of the four species studied here are responding accordingly and will probably continue to change. However, predicting how tropical species will react in face of environmental change is still a big challenge [46]. The lack of consistent patterns of phenological responses among the four species (e.g. flowering delayed in *Miconia quinquedentata* and remained unaltered in *M*. *acutiflora*; fruiting delayed in *Pleroma clavatum* and advanced in *M*. *acutiflora*) suggests that climate change has unequal effects across the Atlantic Forest and the tropical region as a whole. This highlights the urgent need for further research in the field, in order to understand and forecast the ecological implications of these changes in global ecosystem processes. The combination of techniques used here (e.g. massive herbarium information, circular statistics applied to delimited time intervals, climate layers) has proved to be promising for studies on tropical plant phenology.

## Supporting information

S1 Fig Distribution of specimens flowering (A) and fruiting (B) per month. Number of specimens flowering and fruiting per month between 1920 and 2018 (Miconia acutiflora: n = 196; M. quinquedentata: n = 183; Pleroma clavatum: n = 102; P. trichopodum: n = 186).
(TIF)

S2 Fig Mean monthly precipitation and temperature in three time intervals (1920–79, 1980–99, 2000–16) in the northern- and southern-most geographic coordinates used in this study. Climatic data were obtained from CHELSAcruts data series.
(TIF)

S3 Fig Results from the contingency tests showing the distribution of flowering and fruiting specimens in each month (numbered as 1 to 12, from January onwards) and each time interval (t1: 1920–79; t2: 1980–99; t3: 2000–18) of *Miconia acutiflora*, *M*. *quinquedentata*, *Pleroma clavatum* and *P*. *trichopodum*. Circle sizes are proportional to the number of specimens in each month and time interval. Color scale represents Pearson’s residuals: blue and red show respectively positive and negative association between month and time interval.
(TIF)

S1 Table Total number of specimens. Number of specimens used in this study of *Miconia acutiflora*, *M*. *quinquedentata*, *Pleroma clavatum* and *P*. *trichopodum* in each one of the proposed time intervals.
(DOCX)

S2 Table Results from ML analysis. The maximum-likelihood analysis was performed to determine best-fit models of distribution for flowering and fruiting events of *Miconia acutiflora*, *M*. *quinquedentata*, *Pleroma clavatum* and *P*. *trichopodum* for each time interval.
(DOCX)

S3 Table Results from ANOVA. The one-way ANOVA analyses were performed to access variation in precipitation, minimum and maximum temperature among time intervals (1920–1979, 1980–1999, 2000–2016) in both the northern- and southernmost geographic coordinates used in this study. Significant *p-*values are in bold.
(DOCX)

S4 Table Results from regression analysis using the exact coordinates (where collections took place). (XLSX)

S1 File R code for Watson’s U^2^ test for grouped data. The following code adds the option for grouped data to the “watson.two.test” function in *circular* package [38].
(PDF)
