# Supplementary figures and images for: Phenological responses to climate change based on a hundred years of herbarium collections of tropical Melastomataceae
Source: PLoS One. 2021 May 7;16(5):e0251360. doi: 10.1371/journal.pone.0251360 (PMC8104365; doi:10.1371/journal.pone.0251360)

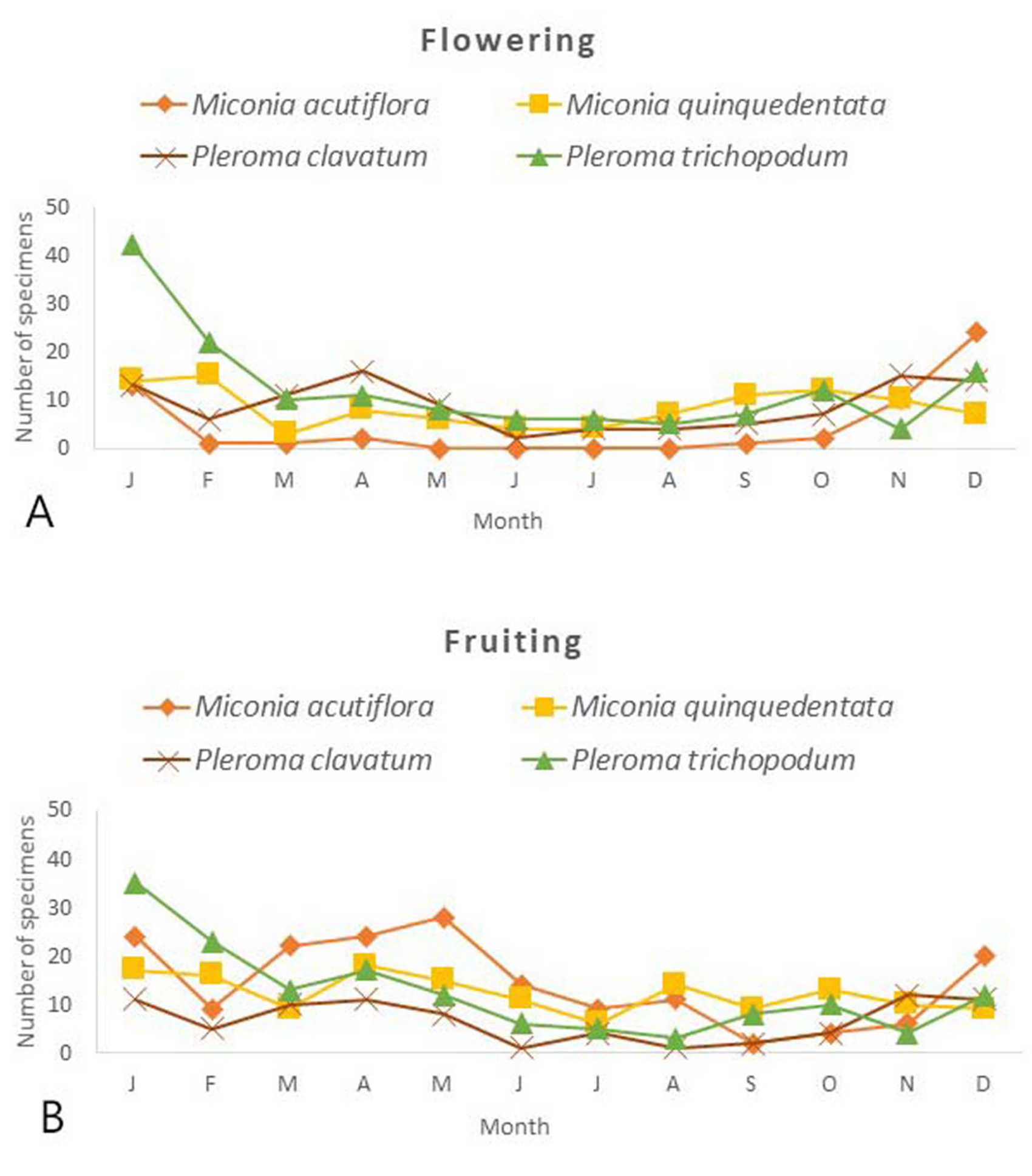

Supplement: S1 Fig — Distribution of specimens flowering (A) and fruiting (B) per month. Number of specimens flowering and fruiting per month between 1920 and 2018 (Miconia acutiflora: n = 196; M. quinquedentata: n = 183; Pleroma clavatum: n = 102; P. trichopodum: n = 186). (TIF) [file pone.0251360.s001.tif]

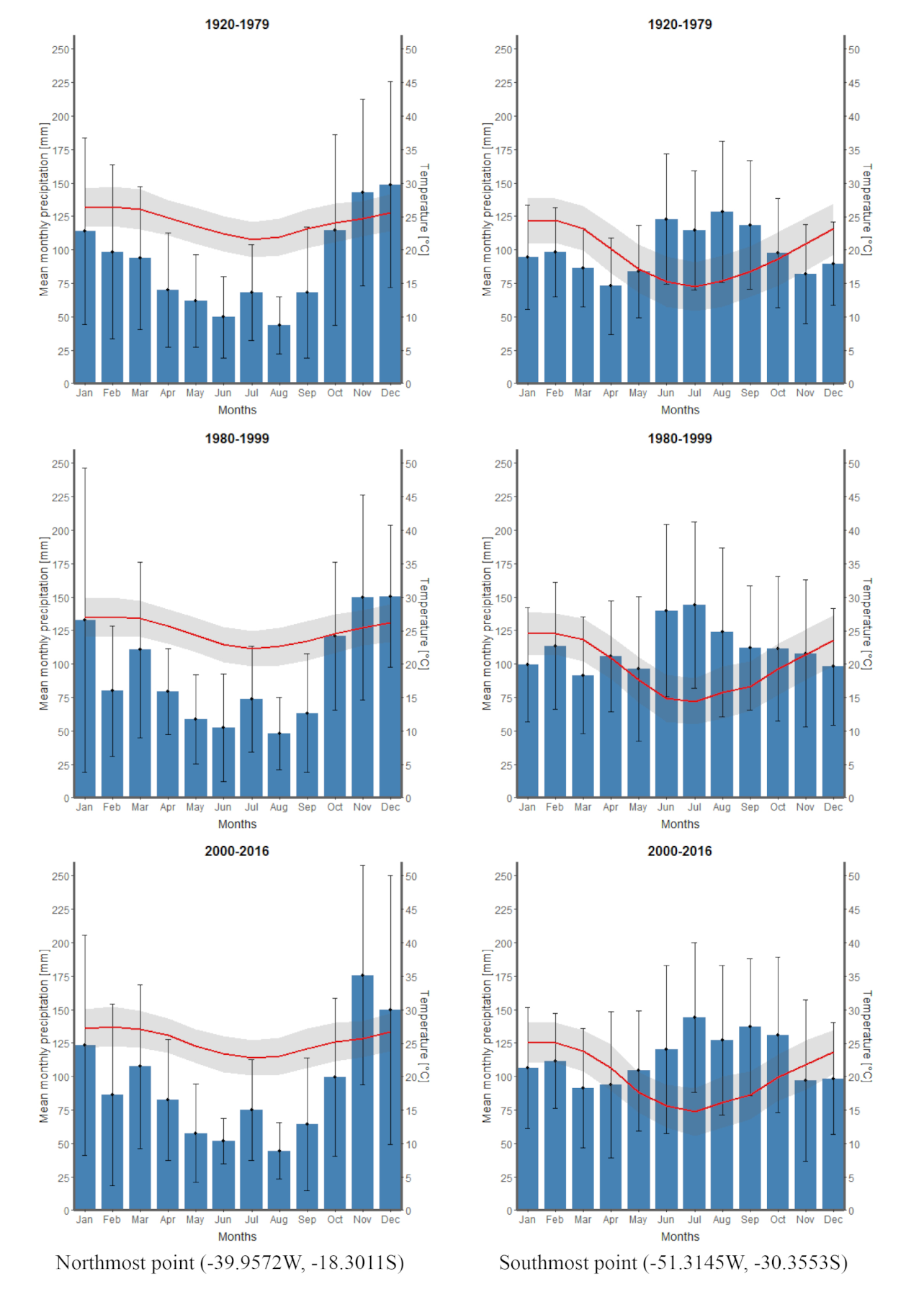

Supplement: S2 Fig — Climatic data were obtained from CHELSAcruts data series. (TIF) [file pone.0251360.s002.tif]

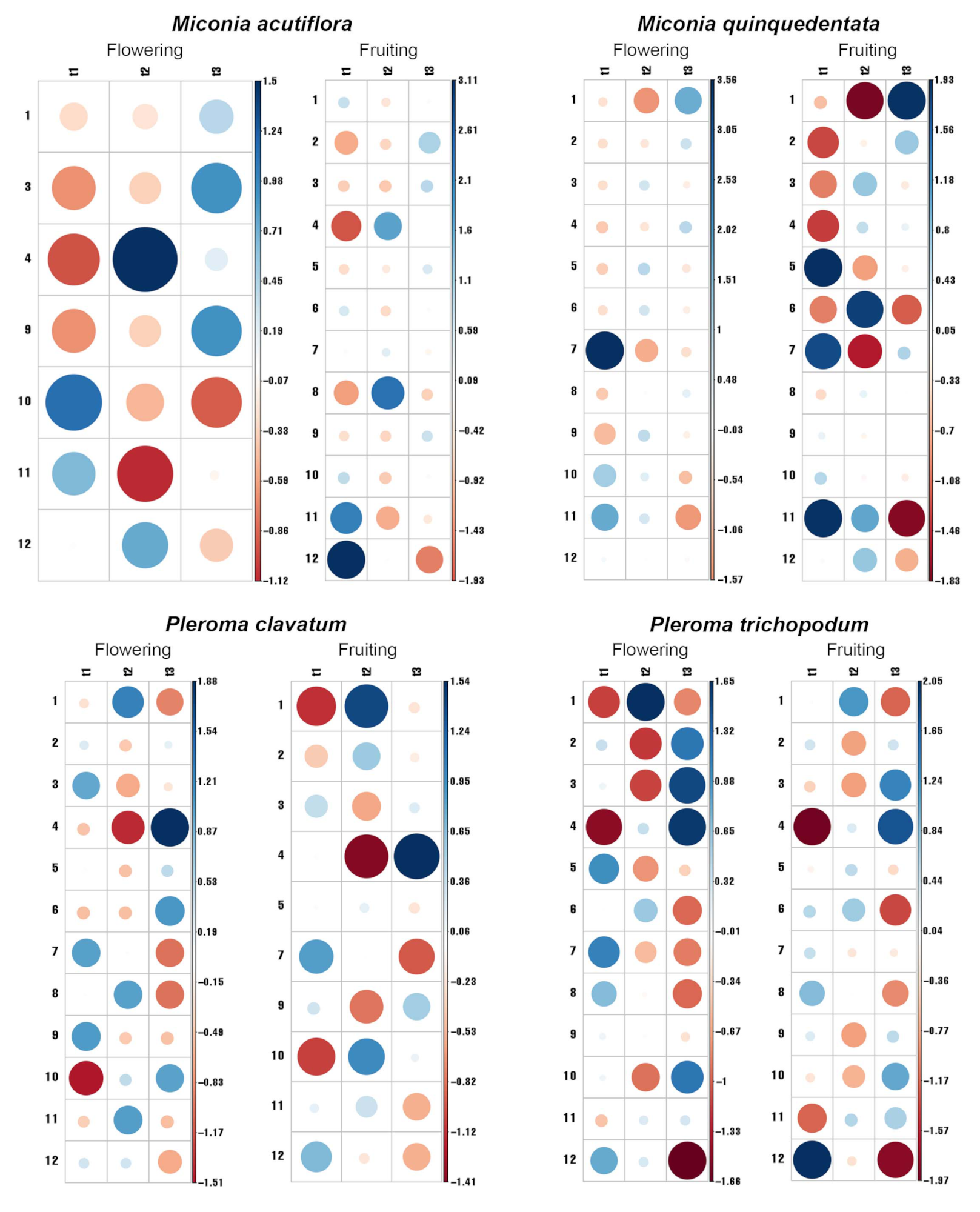

Supplement: S3 Fig — Circle sizes are proportional to the number of specimens in each month and time interval. Color scale represents Pearson’s residuals: blue and red show respectively positive and negative association between month and time interval. (TIF) [file pone.0251360.s003.tif]
