# Supplementary material for: Phenological responses to climate change based on a hundred years of herbarium collections of tropical Melastomataceae
Source: PLoS One. 2021 May 7;16(5):e0251360. doi: 10.1371/journal.pone.0251360 (PMC8104365; doi:10.1371/journal.pone.0251360)
Supplement: S1 Table — Number of specimens used in this study of Miconia acutiflora, M. quinquedentata, Pleroma clavatum and P. trichopodum in each one of the proposed time intervals. (DOCX) [file pone.0251360.s004.docx]

**S1 Table. Total number of specimens.** Number of specimens used in this study of *Miconia acutiflora*, *M. quinquedentata*, *Pleroma clavatum* and *P. trichopodum* in each time interval analyzed.

| **Species/Interval** | **1920-1979** | **1980-1999** | **2000-2018** |
| --- | --- | --- | --- |
| *Miconia acutiflora* | 36 | 42 | 121 |
| *Miconia quinquedentata* | 15 | 73 | 92 |
| *Pleroma clavatum* | 38 | 36 | 35 |
| *Pleroma trichopodum* | 68 | 67 | 54 |
