# Supplementary material for: Phenological responses to climate change based on a hundred years of herbarium collections of tropical Melastomataceae
Source: PLoS One. 2021 May 7;16(5):e0251360. doi: 10.1371/journal.pone.0251360 (PMC8104365; doi:10.1371/journal.pone.0251360)
Supplement: S2 Table — The maximum-likelihood analysis was performed to determine best-fit models of distribution for flowering and fruiting events of Miconia acutiflora, M. quinquedentata, Pleroma clavatum and P. trichopodum for each time interval. (DOCX) [file pone.0251360.s005.docx]

**S2 Table. Results from ML analysis.** The maximum-likelihood analysis was performed to determine best-fit models of distribution for flowering and fruiting of *Miconia acutiflora*, *M. quinquedentata*, *Pleroma clavatum* and *P. trichopodum* for each time interval studied.

| Species | Time interval | Best model | 2nd best model | AICc | delta AICc | model prob. (AICc) |
| --- | --- | --- | --- | --- | --- | --- |
| ***Flowering*** |  |  |  |  |  |  |
| *Miconia acutiflora* | 1920-1979 | M2A | M2C | 32.334 | 10.523 | 0.994 |
|  | 1980-1999 | M2B | M2A | 5.864 | 15.805 | 0.999 |
|  | 2000-2018 | M2A | M2C | 49.078 | 0.591 | 0.425 |
| *Miconia quinquedentata* | 1920-1979 | M2A | M2B | 45.997 | 0.72 | 0.398 |
|  | 1980-1999 | M1 | M3B | 147.030 | 0.132 | 0.16 |
|  | 2000-2018 | M2C | M2B | 153.148 | 0.18 | 0.335 |
| *Pleroma clavatum* | 1920-1979 | M2A | M2B | 120.466 | 0.588 | 0.297 |
|  | 1980-1999 | M2B | M2A | 106.323 | 0.991 | 0.396 |
|  | 2000-2018 | M2C | M5A | 100.790 | 12.561 | 0.995 |
| *Pleroma trichopodum* | 1920-1979 | M2B | M3B | 200.706 | 0.235 | 0.294 |
|  | 1980-1999 | M2C | M2B | 137.111 | 26.681 | 1.000 |
|  | 2000-2018 | M5A | M5B | 81.430 | 0.756 | 0.38 |
| ***Fruiting*** |  |  |  |  |  |  |
| *Miconia acutiflora* | 1920-1979 | M4A | M3B | 79.866 | 0.369 | 0.21087 |
|  | 1980-1999 | M2A | M2C | 125.845 | 0.185 | 0.25647 |
|  | 2000-2018 | M2A | M5A | 351.982 | 2.044 | 0.55069 |
| *Miconia quinquedentata* | 1920-1979 | M1 | M3A | 58.812 | 1.969 | 0.42035 |
|  | 1980-1999 | M1 | M3A | 209.518 | 1.676 | 0.42743 |
|  | 2000-2018 | M2C | M4B | 256.636 | 0.624 | 0.24715 |
| *Pleroma clavatum* | 1920-1979 | M2B | M3B | 172.431 | 1.663 | 0.49226 |
|  | 1980-1999 | M2C | M2B | 134.525 | 17.545 | 0.99954 |
|  | 2000-2018 | M5A | M5B | 145.788 | 0.23 | 0.33906 |
| *Pleroma trichopodum* | 1920-1979 | M5A | M2A | 102.294 | 0.529 | 0.20831 |
|  | 1980-1999 | M2A | M2B | 80.272 | 0.403 | 0.33189 |
|  | 2000-2018 | M2B | M5A | 73.278 | 0.177 | 0.20853 |
