# Supplementary material for: Phenological responses to climate change based on a hundred years of herbarium collections of tropical Melastomataceae
Source: PLoS One. 2021 May 7;16(5):e0251360. doi: 10.1371/journal.pone.0251360 (PMC8104365; doi:10.1371/journal.pone.0251360)
Supplement: S3 Table — The one-way ANOVA analyses were performed to access variation in precipitation, minimum and maximum temperature among time intervals (1920–1979, 1980–1999, 2000–2016) in both the northern- and southernmost geographic coordinates used in this study. Significant p-values are in bold. (DOCX) [file pone.0251360.s006.docx]

**S3 Table. Results from ANOVA.** The one-way ANOVA analyses were performed to access variation in precipitation, minimum and maximum temperature among time intervals (1920-1979, 1980-1999, 2000-2016) in both the northern- and southernmost geographic coordinates used in this study. Significant *p-*values are in bold.

| *Group* | *n* | *Sum* | *Mean* | *Variance* |  | *Variation source* | *SS* | *d.f.* | *MS* | *F-value* | *p-value* | *F critical* |
| --- | --- | --- | --- | --- | --- | --- | --- | --- | --- | --- | --- | --- |
| **Precipitation (lat_min_)** | | | | | | | | | | | | |
| 1920-1979 | 60 | 188315 | 3138.583 | 26829.09 | ANOVA | Among groups | 1038715 | 2 | 519357.6 | 19.15053 | **1.06E-07** | 3.093266 |
| 1980-1999 | 20 | 66662 | 3333.1 | 26648.62 |  | Within groups | 2549257 | 94 | 27119.75 |  |  |  |
| 2000-2016 | 17 | 57299 | 3370.529 | 28751.01 |  | Total | 3587972 | 96 |  |  |  |  |
| **Minimum temperature (lat_min_)** | | | | | | | | | | | | |
| 1920-1979 | 60 | 947.5 | 15.79167 | 0.23322 | ANOVA | Among groups | 13.92679 | 2 | 6.963395 | 31.03611 | **4.47E-11** | 3.093266 |
| 1980-1999 | 20 | 324.94 | 16.24708 | 0.168599 |  | Within groups | 21.09024 | 94 | 0.224364 |  |  |  |
| 2000-2016 | 17 | 285.33 | 16.78382 | 0.257929 |  | Total | 35.01703 | 96 |  |  |  |  |
| **Maximum temperature (lat_min_)** | | | | | | | | | | | | |
| 1920-1979 | 60 | 1388.6 | 23.14306 | 0.227298 | ANOVA | Among groups | 1.833776 | 2 | 0.916888 | 5.063373 | **0.00816** | 3.093266 |
| 1980-1999 | 20 | 465.4 | 23.27 | 0.09895 |  | Within groups | 17.02175 | 94 | 0.181082 |  |  |  |
| 2000-2016 | 17 | 399.7 | 23.51176 | 0.108195 |  | Total | 18.85553 | 96 |  |  |  |  |
| **Precipitation (lat_max_)** | | | | | | | | | | | | |
| 1920-1979 | 60 | 64408 | 1073.467 | 37793.44 | ANOVA | Among groups | 46551.23 | 2 | 23275.62 | 0.514147 | 0.59968 | 3.093266 |
| 1980-1999 | 20 | 22393 | 1119.65 | 53412.66 |  | Within groups | 4255413 | 94 | 45270.35 |  |  |  |
| 2000-2016 | 17 | 18993 | 1117.235 | 63172.44 |  | Total | 4301964 | 96 |  |  |  |  |
| **Minimum temperature (lat_max_)** | | | | | | | | | | | | |
| 1920-1979 | 60 | 1284.6 | 21.41 | 0.177751 | ANOVA | Among groups | 19.26693 | 2 | 9.633465 | 66.27287 | **1.11E-18** | 3.093266 |
| 1980-1999 | 20 | 441.75 | 22.0875 | 0.09958 |  | Within groups | 13.6639 | 94 | 0.145361 |  |  |  |
| 2000-2016 | 17 | 382.83 | 22.51912 | 0.080284 |  | Total | 32.93083 | 96 |  |  |  |  |
| **Maximum temperature (lat_max_)** | | | | | | | | | | | | |
| 1920-1979 | 60 | 1620.4 | 27.00583 | 0.186401 | ANOVA | Among groups | 16.18303 | 2 | 8.091516 | 53.22696 | **3.49E-16** | 3.093266 |
| 1980-1999 | 20 | 551.99 | 27.59958 | 0.095713 |  | Within groups | 14.2898 | 94 | 0.152019 |  |  |  |
| 2000-2016 | 17 | 476.61 | 28.03578 | 0.092099 |  | Total | 30.47283 | 96 |  |  |  |  |
