# Supplementary material for: Phenological responses to climate change based on a hundred years of herbarium collections of tropical Melastomataceae
Source: PLoS One. 2021 May 7;16(5):e0251360. doi: 10.1371/journal.pone.0251360 (PMC8104365; doi:10.1371/journal.pone.0251360)
Supplement: S1 File — The following code adds the option for grouped data to the “watson.two.test” function in circular package [38]. (PDF) [file pone.0251360.s008.pdf]

```
function(data1, data2, ties = F){
  n1 <- length(data1)
  n2 <- length(data2)
  n <- n1 + n2
  data1 <- cbind(sort(data1 %% (2 * pi)), rep(1, n1))
  data2 <- cbind(sort(data2 %% (2 * pi)), rep(2, n2))
  data1_2 <- rbind(data1, data2)
  data.ranks <- order(data1_2[, 1])
  data1_2 <- cbind(data1_2[data.ranks,  ], 1:n)

  if(ties == T)
    {
    angl.interv <- unique(data1_2[1:n,1])

    t1i <- vector(length = length(angl.interv))
    t2j <- vector(length = length(angl.interv))
    for(z in 1:length(angl.interv))
      {
      t1i[z] <- sum(data1_2[1:n,1]==angl.interv[z]&data1_2[1:n,2]==1)
      t2j[z] <- sum(data1_2[1:n,1]==angl.interv[z]&data1_2[1:n,2]==2)
      }

    m1i <- vector(length = length(angl.interv))
    m2j <- vector(length = length(angl.interv))
    m1i[1]<-t1i[1]
    m2j[1]<-t2j[1]
    for(z in 2:length(angl.interv))
      {
      m1i[z] <- m1i[z-1]+t1i[z]
      m2j[z] <- m2j[z-1]+t2j[z]
      }

    m1i_n1 <- m1i/n1
    m2j_n2 <- m2j/n2

    dk <- m1i_n1-m2j_n2
    dk2 <- dk^2
    tk <- t1i+t2j
    tkdk <- sum(tk*dk)
    tkdk2 <- sum(tk*dk2)

    u2 <- ((n1 * n2)/n^2)*(tkdk2 - ((tkdk^2)/n))
    result <- list(statistic=u2,nx=n1, ny=n2, grouped.data = "yes")
    return(result)
    }

  if(ties == F)
    {
    i <- 1:n
    j <- 1:n
    for (z in 1:n)
      {
      i[z] <- sum(data1_2[1:z, 2] == 1)
      j[z] <- sum(data1_2[1:z, 2] == 2)
      }
    d <- j/n2 - i/n1
    dbar <- mean.default(d)
    u2 <- ((n1 * n2)/n^2) * sum((d - dbar)^2)
    result <- list(statistic=u2,nx=n1, ny=n2, grouped.data = "no")
    return(result)
    }
}
```
